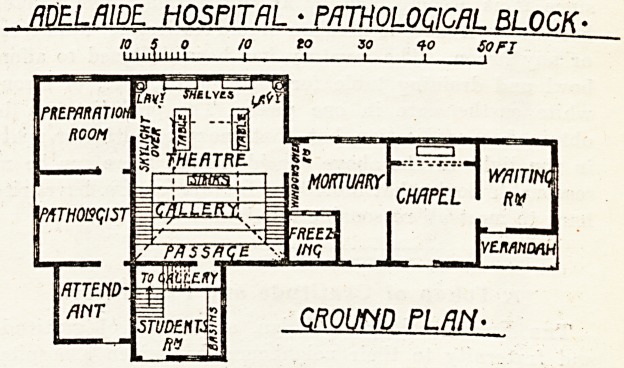# Adelaide Hospital, South Australia

**Published:** 1915-09-25

**Authors:** 


### Adelaide Hospital, South Australia.

This latest addition to the Adelaide Hospital was completed in 1914. It provides a complete department for the work of pathology and the orderly and decent disposal of the dead.

The *post-mortem* theatre is a large room with accommodation for fifty-eight students, and two *post-mortem* tables. The walls of this room and those of the mortuary and chapel are lined to a height of six feet with white tiles, above which is a hard cement. On one side of the theatre is the pathologists' laboratory, having adjacent to it the attendants' room and a preparation room where students work under the pathologist. On the other side is the mortuary, with a freezing chamber for nine bodies, a mortuary chapel, and waiting-room.

The students' entrance is at the back of the theatre, and under the stairs is a lavatory, etc.

## Figures and Tables

**Figure f1:**